# PTFE layer formation during brush electroplating of nickel

**DOI:** 10.1038/s41598-024-71376-5

**Published:** 2024-10-14

**Authors:** L. Isern, S. Impey, H. Almond, S. J. Clouser, J. L. Endrino

**Affiliations:** 1 School of Aerospace, Transport and Manufacturing (SATM), Cranfield University, College Road, Cranfield, Bedfordshire, MK43 0AL UK; 2 SIFCO ASC, 5708 E Schaaf Road, Independence, OH 44131 USA; 3 Universidad Loyola Andalucía, 41704 Dos Hermanas, Seville, Spain

**Keywords:** Surfaces, interfaces and thin films, Materials science, Nanoscience and technology

## Abstract

Brush electrodeposition of Ni/PTFE composite coatings was explored using a nickel high speed solution and polytetrafluoroethylene (PTFE) particles 6–9 μm in diameter. A novel bilayer-like, partially intercalated structure was produced, consisting of a rough nickel sublayer covered by an outer, compact, smooth PTFE layer. The study of the coating growth revealed that the PTFE particles bind together on the nickel coating valleys and grow until all the surface is covered by a polymer layer without the need of a baking stage. The resulting coating presents a hydrophobic surface with a low coefficient of friction (0.10) and higher corrosion resistance to salt spray testing than the nodular nickel coating. The coatings were produced using an aqueous nickel plating solution, where the hydrophobic PTFE particles were suspended using different substances: cetrimonium bromide (CTAB) cationic surfactant, isopropyl alcohol premixed with the particles, and ethanol premixed with the particles. High concentrations of the suspending products were detrimental for the deposition process, but optimal values of 0.1 g/l, 3 ml/l and 3 ml/l respectively were found. All compounds successfully suspended the PTFE particles and both alcohols produced the Ni/PTFE coating described before, but the CTAB failed to co-deposit the polymer.

## Introduction

The deposition of composite coatings by electroplating and electroless processes requires the addition of particles in metallic coatings, which improves the composite properties beyond the original matrix. For instance, the addition of carbides increases wear resistance^1^, solid lubricants decrease friction^2^ and some metals and carbides increase corrosion resistance^3,4^. To produce the composite coating, particles are suspended in the plating bath and metal ions deposition induces particle co-deposition^5^. However, particles such as polytetrafluoroethylene (PTFE) present an additional challenge due to their hydrophobicity in aqueous media. To accomplish particle suspension, surfactants are added to the solution. Surfactants are organic molecules with two ends: one hydrophobic and one hydrophilic. The hydrophobic end attaches to the nearby PTFE particles, surrounding them and creating a screen. The hydrophilic end is then free to interact with water molecules, effectively suspending the PTFE particles within the aqueous solution. The use of surfactants often requires agitation, ultrasonic and mechanical homogenisers for a fine, disperse suspension of PTFE^6,7^.

There are different types of surfactants depending on the polarity of the hydrophilic end: cationic ( +), anionic ( −), non-ionic (no charge) and amphoteric (both + and −). The effectiveness of different surfactants for suspending PTFE particles in plating processes vary depending on the deposition method used. For electroless deposition, non-ionic surfactants are preferred as they are more effective in producing a suspension that is more stable over time and at higher temperatures. Meanwhile, anionic surfactants rapidly lose effectiveness over time and cationic surfactants hold the middle ground between the two^8^. On the other hand, the electroplating deposition is conditioned by the potential differential established between the electrodes. In this case, cationic surfactants are preferred, as PTFE particles are electrostatically charged by the adsorption of the surfactants and studies show that the amount of co-deposited PTFE is controlled by the zeta potential of the particles^9^. With cationic surfactants, PTFE particles share the same positive charge as the metal ions being deposited. Additionally, the quality of the electrodeposited coating is reported to be better with cationic and non-ionic surfactants^10^. Many studies reveal good performance of cationic families of the hexadecyltrimethylammonium bromide (e.g. CTAB)^7,10,11^ and the cetrimonium iodine^12–14^ for PTFE particle sizes from the nanometric scale to 9 μm. Other studies use commercially available PTFE suspensions containing nano-sized particles (50–200 nm) for electroless^10^, brush plating^15,16^ and jet electroplating^14^ processes.

Hence, the PTFE suspension and co-deposition is not a simple task. The rewards are the properties of the resulting coatings. Typically, a metal matrix composite is formed with metal ions and a dispersed phase of PTFE particles. Hydrophobicity and superhydrophobicity are common features of the composite coating surfaces^7,12,13^, obtaining water contact angles greater than bulk PTFE. Compared to the original matrices (e.g. pure nickel), the coefficient of friction of the composites is lower^17,18^ and both the sliding wear resistance^17–19^ and corrosion resistance are higher^10,14,20^. The hardness has been reported to increase in electroless coatings, especially after heat treatment^17^, although tank and jet electroplated coatings show a hardness decrease following the rule of mixtures between matrix and particles^7,14^. The benefit in properties make the PTFE composite coatings ideal for applications in corrosive environments and sliding wear, and also have use as anodes in Ni-MH batteries^21^.

The purpose of this work is to evaluate the effectiveness of using a cationic surfactant (CTAB) to deposit Ni/PTFE coatings in comparison with the use of alcohol. PTFE particles form suspensions in both isopropyl alcohol and ethanol, which in turn are miscible in water. The research covers the performance of a premixed alcohol/PTFE suspension that is added to the plating solution. The coatings so produced are characterised to determine their morphology and growth, and to assess their hydrophobicity, friction properties and corrosion resistance.

## Experimental methods

### Brush electroplating equipment

Three elements are needed for the brush plating: a rectifier to produce and control a voltage, an anode covered by a brush to transmit the voltage to the samples and hold the plating solution, and a solution circulation system. A pe2010 was used, a manufactured by plating electronic (Germany). Inert graphite blocks and iridium oxide-coated titanium meshes were used as anodes. To cover the anodes, two different 3 M Scotch-Brite™ fabrics were used: abrasive red (code 7447)^22^ and non-abrasive white (7445)^23^. The colours refer to their appearances and will be used thorough the paper for simplicity. The solution was circulated using a system designed for metal matrix composite (MMC) deposition and minimise particle sedimentation. The system is described and tested in a previous study^24^ and consists of a steep-walled funnel to collect the solution, a magnetic-stirred reservoir to keep the particles in suspension, and a peristaltic pump to recirculate the solution.

### Brush electroplating conditions

Deposition of Ni and Ni/PTFE coatings on mild steel substrates (S275JR defined in standard BS EN 10025-2: 2011) was undertaken. The Ni was deposited following the SIFCO Process® using the appropriate solutions for cleaning, activating the surface, depositing a 2-µm bonding layer of nickel, followed by further nickel deposition (see Table 1). The PTFE was added in powder form (diameter 6–9 µm, supplied by Goodfellow, UK) to plating solution in quantities ranging from 20 to 30 g of PTFE per litre of solution. To overcome the hydrophobicity of the PTFE particles, different compounds were used:

- Isopropyl alcohol (IPA) and ethanol supplied by Fisher Scientific (UK) were added in the quantity of 3 ml per gram of PTFE. The alcohols were premixed with the PTFE powders and stirred for 5 min before being added to the plating solution.
- N,N,N-Trimethylhexadecan-1-aminium bromide (preferred IUPAC name), also known as Hexadecyl(trimethyl)azanium;bromide, cetrimonium bromide, hexadecyltrimethylammonium bromide and CTAB, supplied by Sigma-Aldrich (UK) was added in quantities ranging from 0.1 to 4 g per litre of plating solution. The mix of CTAB with the plating solution was subjected to ultrasonic agitation for 10 min and then magnetically stirred for 3 h. Then, the PTFE powder was added and stirring continued for 16 h at 1000 rpm to guarantee blending.

**Table 1:** Commercially available solutions and plating conditions used in the brush plating process, manufactured by SIFCO ASC (US).

Step	Solution	Composition	Anode material	Conditions
Surface preparation	Electroclean	3% Sodium Citrate 2% Sodium Hydroxide	Graphite	15 V
	Etching No.2	11% Sodium Chloride 2% Hydrochloric Acid	Graphite	9 V, reverse current
	Etching No.3	15% Sodium Citrate 6% Citric Acid	Graphite	15 V, reverse current
Pre-plating	Nickel Special	21% Nickel Sulfate 4% Citric Acid 2% Hydrochloric Acid 2% Acetic Acid	Graphite	10 V, 2 µm thick
Plating	Nickel High Speed	11% Nickel Sulfate 10% Ammonium Formate 5% Ammonium Citrate 4% Ammonia Hydroxide	Graphite and iridium oxide-coated titanium mesh	37–124 A/dm^2^

Steel substrate areas 30 × 30 mm and 50 × 50 mm were masked off and coated, with a coating thickness controlled by measuring a total charge applied of 38 A∙min with the rectifier’s Ampere-hour counter, which corresponds to 60 µm on a 900 mm^2^ area. Different current densities were used (37, 75 or 124 A/dm^2^). After deposition, all samples were rinsed in deionised water and ultrasonically cleaned in IPA.

### Coating characterisation

A FEI XL30 ESEM scanning electron microscope (SEM) was used to examine the coating morphology. Images were taken using secondary and back-scattered electrons detectors with 20 kV at a working distance of 8–18 mm. Composition maps were obtained using an energy dispersive x-ray (EDX) spectroscopy module from the SEM with Oxford Instruments Aztec software. Roughness measurements were obtained from 3D maps of coating surface areas of 640 × 480 µm using an Olympus Lext OLS3100 confocal laser scanning microscope (CLSM). The crystalline structure of the coatings was determined using a Philips D5005 X-ray diffractometer with Cu K_α_ radiation at 20 kV. The hydrophobicity of the samples was assessed by depositing small water droplets on the surfaces (approximate volume 5 μl) and, after a stabilisation period of 30 s, measuring the contact angle with an optical tensiometer (Attension Theta Lite, Biolin Scientific). The coefficient of friction of the coatings was measured against chrome steel balls (Ø 5 mm) using a Teer ST3001 scratch tester; a progressive, increasing load of 5-100N was applied across 10 mm. Finally, corrosion tests were carried out in a salt spray chamber Q-Fog Cyclic Corrosion Tester, Model # Q-FOG / CCT 600, following the standard practice of ASTM B117.

## Results

### Ni/PTFE suspensions and co-deposition

The first challenge in depositing Ni/PTFE composite coatings by brush electroplating is to obtain a suspension of the PTFE particles in the aqueous plating solution. The use of surfactants or other compounds is needed to overcome the hydrophobicity of the PTFE, so the particles can be suspended in the solution and later co-deposited with the nickel ions. This section reports the use of two alcohols and one anionic surfactant (CTAB) and compares them to the deposition of pure nickel coatings and the use of PTFE powder without suspending agents.

#### Pure nickel coatings

**Fig. 1:**
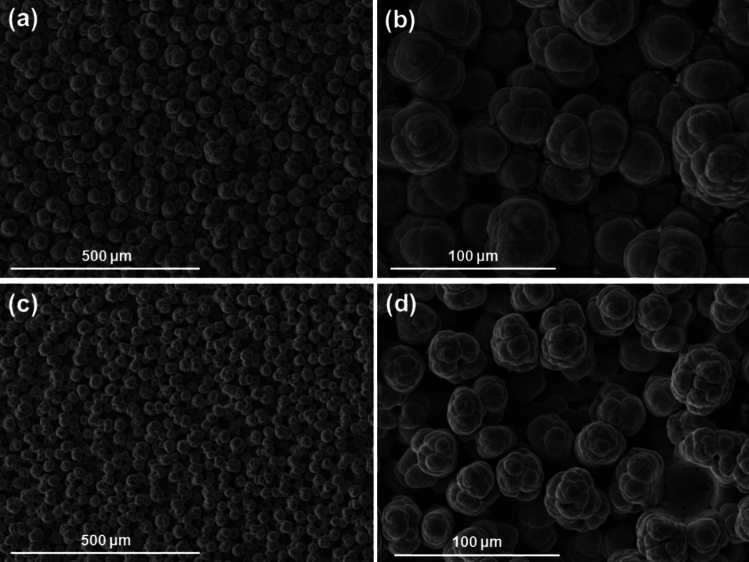
SEM surface images of two pure nickel coatings deposited with a white brush material. (**a**,**b**) show two magnifications of a sample produced with a current density of 75 A/dm^2^ (**c**,**d**) correspond to a coating deposited at 124 A/dm^2^.

Pure nickel was deposited as baseline for comparison from nickel high speed solution, using current densities of 37, 75 and 124 A/dm^2^ and red and white brush materials. Figure 1 shows the globular structure formed as nickel grows when using the white brush material. This non-abrasive fabric produces a rougher finish than abrasive red, while increasing current density (Fig. 1c,d) also produces rougher surfaces with globules that are slightly smaller but more branched and less spherical than low current densities (Fig. 1a,b).

#### No surfactant, severe stirring

Production of Ni/PTFE composite was attempted without the use of a suspension agent. PTFE powder was added in the plating solution in quantities of 33–50 g/l, but remained floating in the water and the solution circulation system could not transport it. Magnetic stirring (approx. 700 rpm) allowed the PTFE particles to sink to the circulation inlet and be transported to the sample surface.

**Fig. 2:**
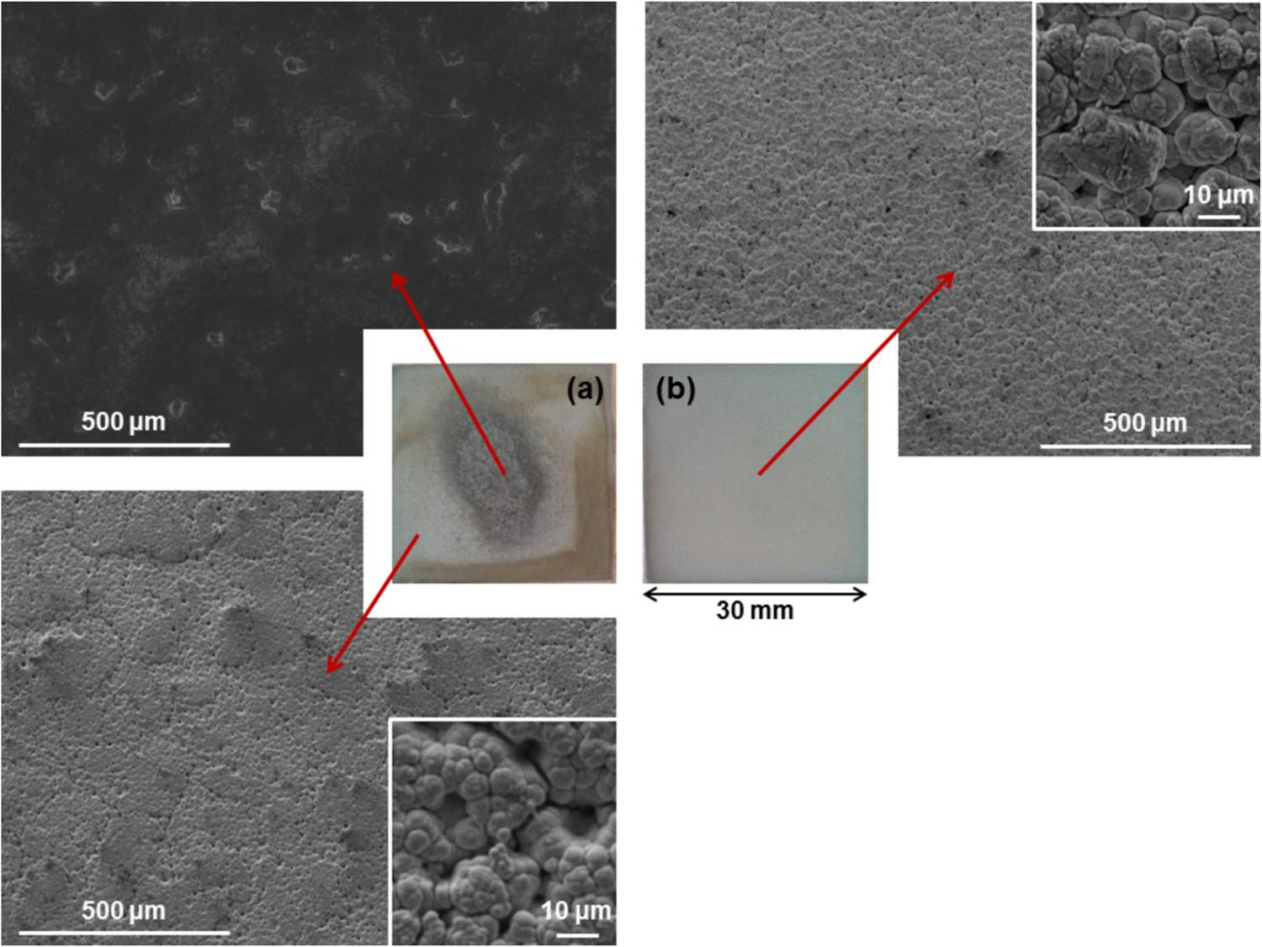
Surface images from Ni/PTFE coating without the use of surfactants. In the centre, two macro images of the coatings, (**a**) produced with white brush and 124 A/dm^2^ current density and (**b**) produced with red brush and 75 A/dm^2^. Around the edges, SEM images taken from the regions indicated with the red arrows are given with higher magnifications in the corners.

The use of white brush and a current density of 124 A/dm^2^ resulted in a non-homogeneous coating, as shown in Fig. 2a. The central area is covered by a PTFE top layer and a nickel sub-layer, whereas the peripheral area is covered by a nickel coating only, no traces of PTFE. Hydrophobicity was noticed at the central area during plating, as it expelled the aqueous solution.

Samples produced with red brush and 75 A/dm^2^ failed to co-deposit the PTFE and produced a pure, single nickel layer (Fig. 2b). EDX and XRD analysis confirmed that no traces of fluorine or PTFE are present. The coating morphology closely relates that of pure Ni coatings under the same plating conditions: a globular structure accentuated in coatings produced by non-abrasive brushes and high current densities, with pores from hydrogen evolution.

#### Hexadecyltrimethylammonium bromide (CTAB)

The cationic surfactant CTAB was added to the nickel sulphate-based plating solution in quantities of 0.1, 0.5, 3 and 4 g per litre of solution. After a thorough blending process, all ranges of CTAB concentrations successfully suspended 20 g/l of PTFE powders in the plating solution. Eight combinations of electroplating parameters were used (37, 60 and 75 A/dm^2^; red and white brush fabrics) to deposit 16 coatings (two per combination). The resulting coatings are a single, pure-Ni layer, failing to incorporate the PTFE powders. Neither surface microphotographs, EDX nor XRD showed the presence of any amount of PTFE.

**Fig. 3:**
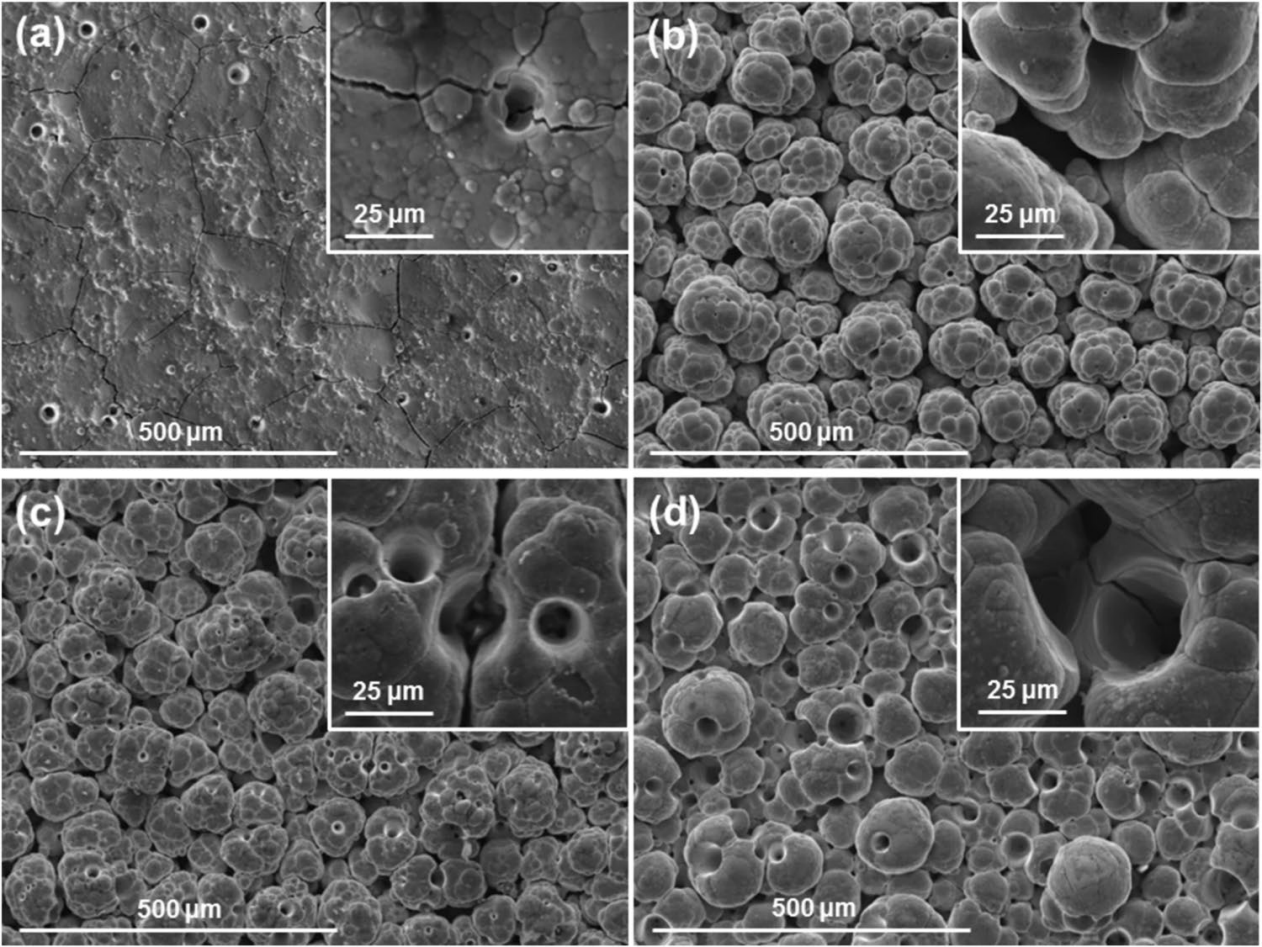
SEM surface images with high magnification detail of Ni coatings produced with white brush and different current densities and CTAB concentrations of: (**a**) 37 A/dm^2^ and 3 g/l; (**b**) 75 A/dm^2^ and 0.1 g/l; (**c**) 75 A/dm^2^ and 0.5 g/l; (**a**) 75 A/dm^2^ and 4 g/l.

**Fig. 4:**
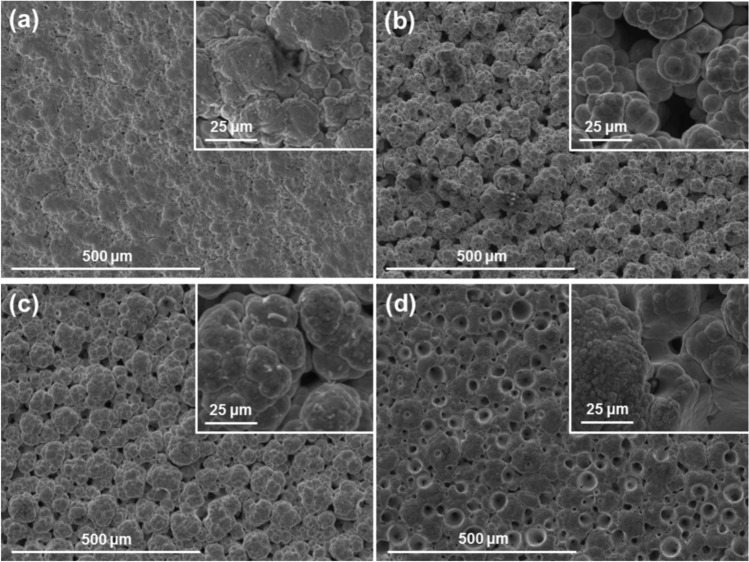
SEM surface images with high magnification detail of Ni coatings produced with red brush and different current densities and CTAB concentrations: (**a**) 37 A/dm^2^ and 3 g/l; (**b**) 75 A/dm^2^ and 0.1 g/l; (**c**) 75 A/dm^2^ and 0.5 g/l; (**a**) 75 A/dm^2^ and 4 g/l.

In all cases, the typical nickel globular structure is present. The surface morphology of the coatings is heavily influenced by both the plating parameters used and the concentration of surfactant present in the bath, as seen in Figs. 3 and 4. The effect is analogous to pure nickel plating: non-abrasive white brush produces large defined globules, while abrasive red brush produces smooth, compact coatings with small globules. Additionally, high current densities produce coatings with more pores and rougher surfaces. Higher concentrations of CTAB surfactant produce coatings with more spherical, less branched globules, and pores in greater quantities and bigger in size than pure nickel high speed solutions. The CTAB also increased the solution resistivity, thus for higher concentrations of CTAB the voltage had to be raised from 9.5–11.5 V to 20 V in order to achieve the same current density of 75 A/dm^2^.

#### Isopropyl alcohol (*IPA*)

The hydrophobic PTFE particles prevent the formation of a suspension in water-based solutions. Nevertheless, PTFE can be suspended in other liquids, such as isopropyl alcohol (IPA), which is miscible in water. An IPA/PTFE suspension was initially formed and then added into the aqueous-based plating solution, where it successfully suspended as small flakes or particle agglomerations. Several ratios of IPA/PTFE were tested from 1 to 10 ml of IPA per gram of PTFE, resulting in several textures from a very dense gel with high agglomeration to a very dilute suspension that produced dissociation of the plating bath in two phases. 3 ml/g was selected as the optimal ratio, and 20 g/l of PTFE was added to the plating solution. Deposition covered areas of 30 × 30 mm and extended for 38 A·min (which, in the case of pure nickel, would deposit a 60 μm thick coating). The coatings described in this section were produced with white brush and current density of 37 A/dm^2^. Additionally, other samples (not discussed here) were produced at higher current densities (65–75 A/dm^2^), obtaining the coating structure as described below.

Soon after deposition process started (1–3 A·min), two phenomena were noticed: i) the resistivity of the system increased and ii) the edges of the plating area showed some signs of hydrophobicity. The voltage had to be increased to maintain high current densities and, due to limitations on the voltage output of the rectifier, samples were deposited at the lower current density (37 A/dm^2^). The second phenomenon, hydrophobicity, was noticed by the quick ejection of the plating solution from the edges of the area to be plated. The areas affected by hydrophobicity started at the coating edges and extended to the centre over time, making the whole surface hydrophobic after 3–7 A·min. Nonetheless, the voltage stabilised after this initial stage and the plating process appeared to carry on without further issues. Finally, once the deposition process finished, a small quantity of PTFE particles remained floating; IPA had to be added to suspend them again before the solution could be re-used.

**Fig. 5:**
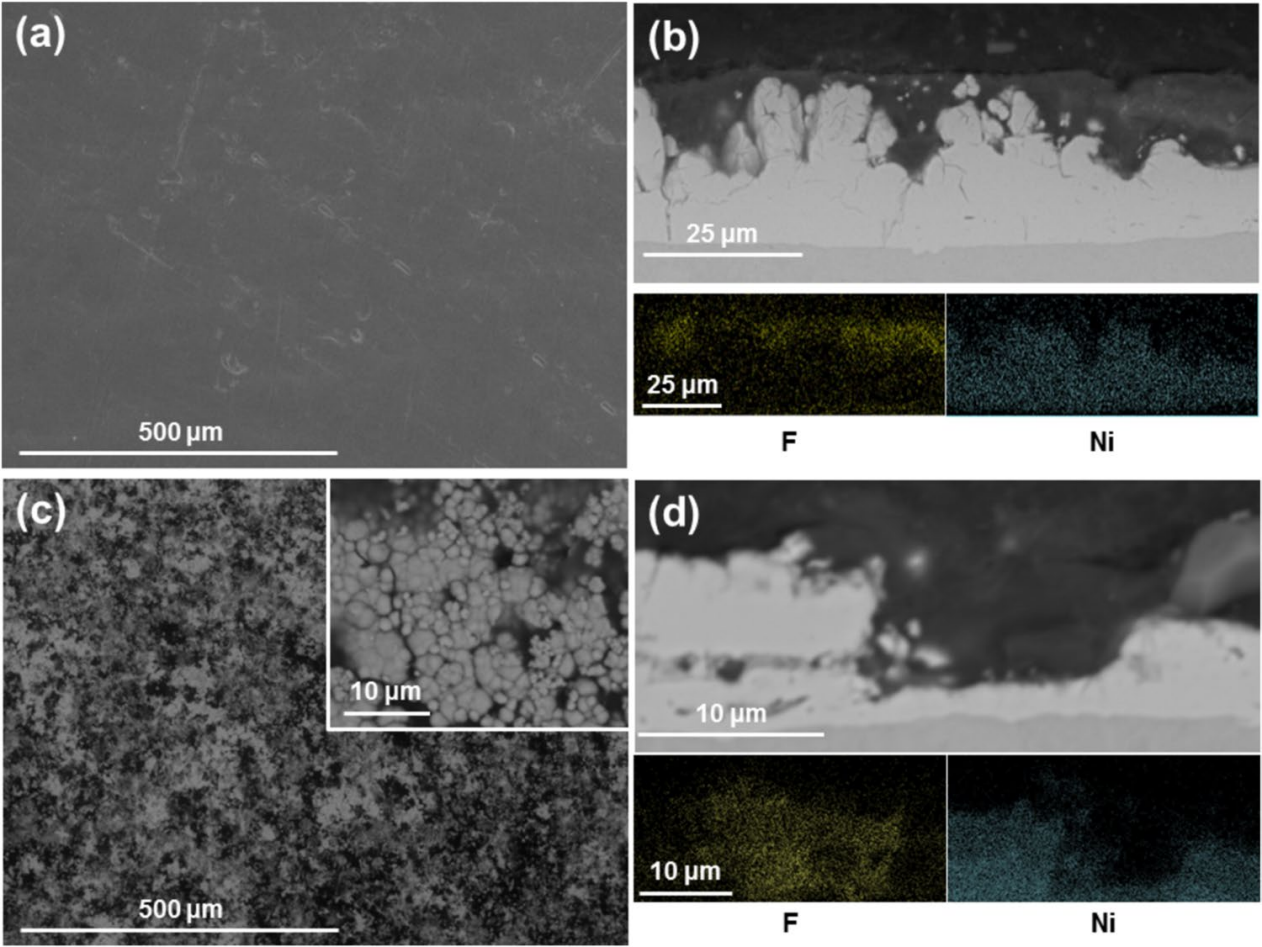
SEM images and EDX mapping of Ni/PTFE coatings produced using IPA as PTFE suspension agent, a current density of 37 A/dm^2^ and white brush. (**a**) corresponds to a secondary electrons image of a thick coating surface, whereas (**b**) is a cross-section (BSE) of (**a**), together with EDX mapping of F (yellow) and Ni (blue). (**c**) is a thin coating surface (BSE), with (**d**) the cross-section (BSE) and EDX mapping of (**c**).

Surface inspection using the environmental mode of the SEM (incorporating 0.7 Torr H_2_O vapour pressure) allows the visualisation of non-conductive samples, avoiding excessive charging of the images. Micrographs of fully formed coatings reveal a dense, smooth surface devoid of any nickel globules (Fig. 5a). EDX measurements at the surface show a concentration of F and C at a F/C ratio of 70/30 (the theoretical ratio of PTFE is 67/33), with Ni peaks representing < 1 at.% of the surface composition in all measurements. Cross-sections of the samples were cut and revealed a PTFE top layer covering a Ni sublayer (Fig. 5b). The nickel layer conserves the typical characteristics and globular structure of nickel high speed coatings, whereas the top PTFE layer totally covers the globules and has a smooth surface, which was also observed by visual inspection and under a bifocal microscope. EDX mapping confirms the identification of the elements forming the layers and the distinction in location of F and Ni atoms, as shown at the foot of Fig. 5b.

One deposition was stopped just after 100% of the surface of the coating became hydrophobic (at 3 A·min) to analyse the coating growth at earlier stages. Surface inspection of the sample revealed a surface with a similar globular nickel structure, albeit at smaller scale, with the deeper valley areas covered by a PTFE layer (Fig. 5c); the back-scattered electron (BSE) detector gave good contrast between both materials. Cross-sections of the coating showed the formation of both layers and EDX mapping (Fig. 5d) confirmed significant differences in elemental composition between layers. The top layer is composed of F and C with a 70/30 ratio, while the bottom layer contains only Ni. The coating was significantly thinner than the previous samples, with a thickness of 9 ± 3 μm compared to the previous 25 ± 3 μm. The nickel layer is also much thinner, which suggests further growth.

#### Ethanol

The use of ethanol as suspension agent produced many similarities to that of IPA. A premix of ethanol and PTFE was successfully suspended in the plating solution, with an ideal ratio of agent/PTFE of 3 ml/g. Plating conditions with a white brush and a current density of 37 A/dm^2^ on a 30 × 30 mm area for 38 A·min were used. The same two phenomena were observed during deposition: increased resistivity and hydrophobicity starting from the edges at 1–3 A·min, covering the plating area by 5–10 A·min. As with IPA, once the deposition process finished, a small quantity of PTFE dropped from suspension and more ethanol had to be added.

**Fig. 6:**
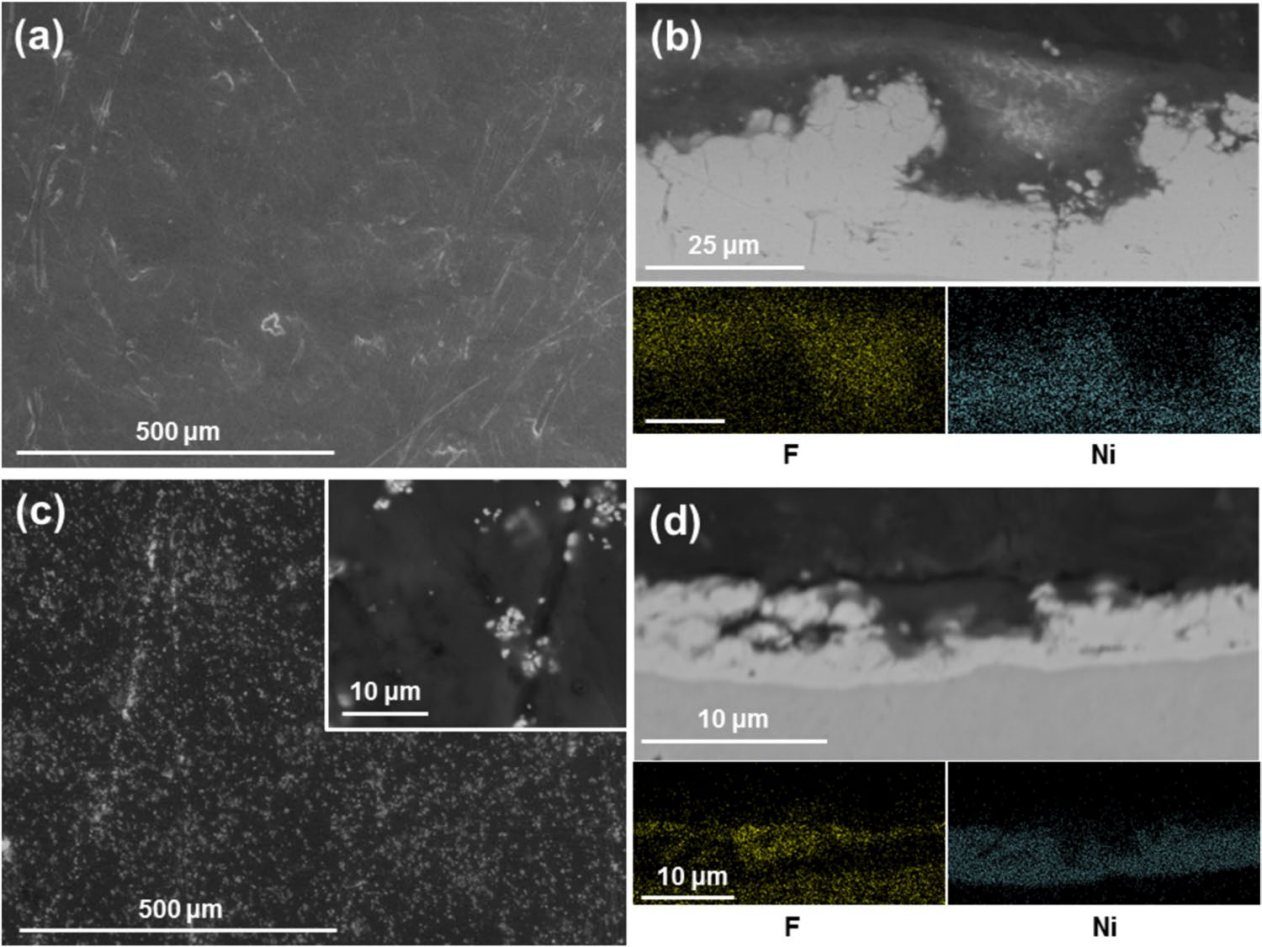
SEM images and EDX mapping of Ni/PTFE coatings produced using ethanol as PTFE suspension agent, a current density of 37 A/dm^2^ and white brush. (**a**) corresponds to a secondary electrons image of a thick coating surface, whereas (**b**) is a cross-section (BSE) of (**a**), together with EDX mapping of F (yellow) and Ni (blue). (**c**) is a thin coating surface (BSE), with (**d**) the cross-section (BSE) and EDX mapping of (**c**).

The coating morphology was as with IPA (Fig. 6a,b). The surface of the coating appears flat and featureless. Cross-sections reveal the double-layered structure of the coating, with PTFE being in the top layer and a globular nickel layer underneath. EDX mapping confirmed the composition of both layers. One deposition was interrupted after 100% surface hydrophobicity at 5 A·min (Fig. 6c,d). In this coating, the tips of the nickel globules are visible with the valleys fully covered with PTFE, more so than for the IPA. The difference in thickness from the IPA sample is probably due to the coating process being interrupted at a later stage. The interrupted coating is significantly thinner with a thickness of 7 ± 1 μm compared to the 26 ± 6 μm of the 38 A·min coatings.

### Characterisation of Ni/PTFE coatings

The morphology of all the nickel based coatings is described in the previous section. Additional characterisation was undertaken on Ni/PTFE to quantify further properties of the coating.

#### Microstructure

**Fig. 7:**
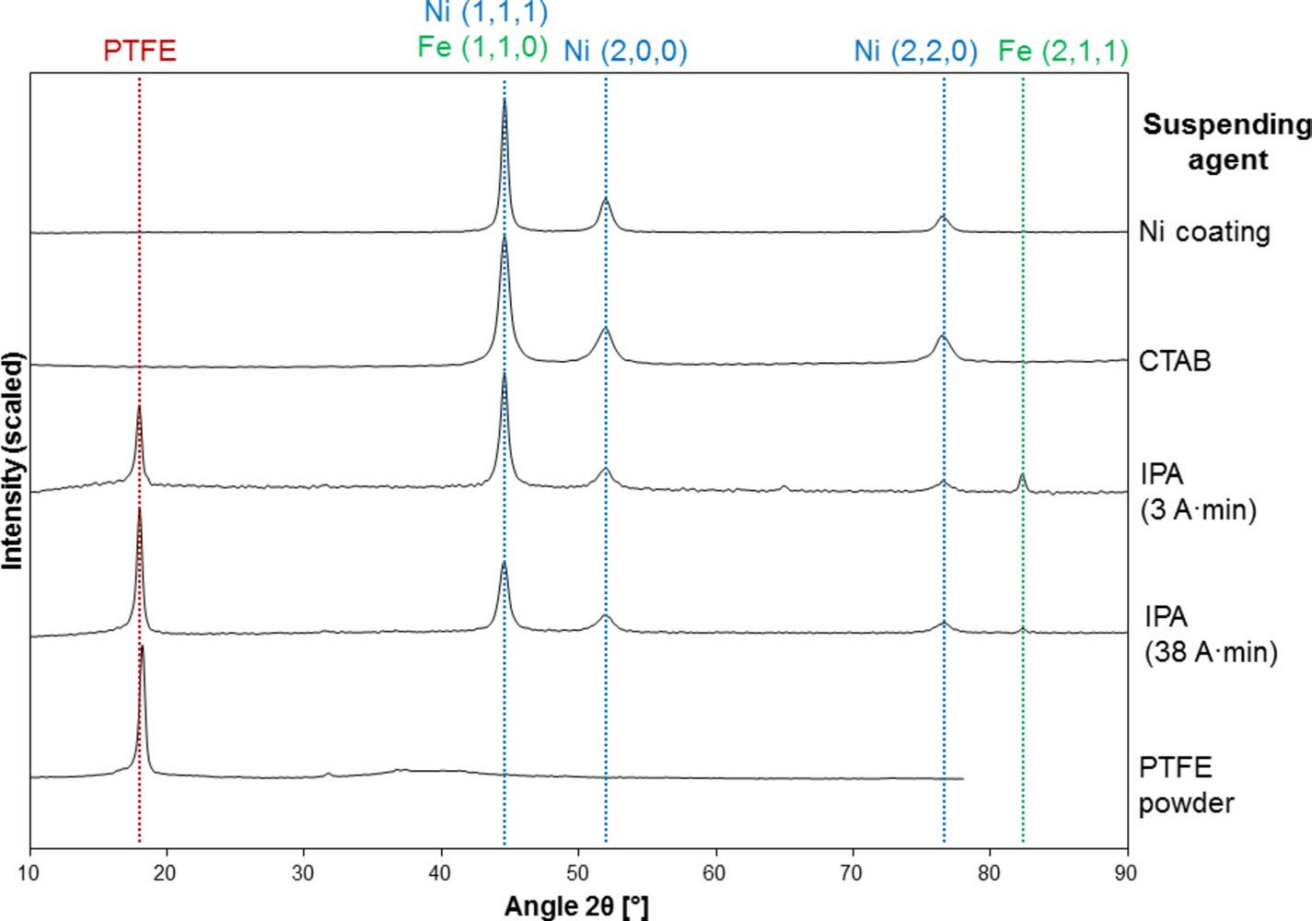
XRD patterns of Ni and Ni/PTFE coatings corresponding, from top to bottom, to a pure Ni coating, a Ni coating produced from a PTFE suspension with CTAB, a Ni/PTFE coating produced with IPA at 3 A·min, a Ni/PTFE coating produced with IPA at 38 A·min, and as-received PTFE powder.

EDX mapping confirmed the presence of F and C atoms on the outer layer of the coating, and XRD patterns complement this. The nickel high speed coating and PTFE powder patterns from the material used in this research match a nickel FCC and crystalline polytetrafluoroethylene spectra in the International Centre for Diffraction Data (ICDD) database, 04–0850 and 47–2217 respectively. Examples of the Ni/PTFE coatings are given in Fig. 7.

Ni coatings plated with the CTAB surfactant follow the same pattern as the pure Ni coatings, without any traces of crystalline PTFE incorporation. The relative intensities of the Ni peaks shift slightly, hinting at a small change in crystal orientation: the relative intensity of the peaks at 44.6°, 51.9°, 76.5° were 1, 0.23, 0.11 for the nickel high speed coating, while the addition of CTAB shifted them to 1, 0.24, 0.18, which represents a significant increase of the (2, 2, 0) peak. The full-width at half-maximum (FWHM) values of the peaks also changed, those of CTAB (0.9, 1.3, 1.3) are appreciably larger than the reference nickel (0.5, 0.9, 1.0).

The patterns of the Ni/PTFE coatings from IPA are a combination of Ni coating and PTFE powder patterns, plus a peak at 82.3° corresponding to the (2,1,1) plane of iron BCC, probably from the substrate. The early-growth samples show stronger Ni and Fe peaks linked to substrate contribution, whereas the thicker coatings have the main peak at 18.0° corresponding to PTFE and the Fe peak is barely noticeable. Finally, the Ni/PTFE coatings produced with ethanol show similar spectra to those with IPA.

#### Hydrophobicity

**Fig. 8:**
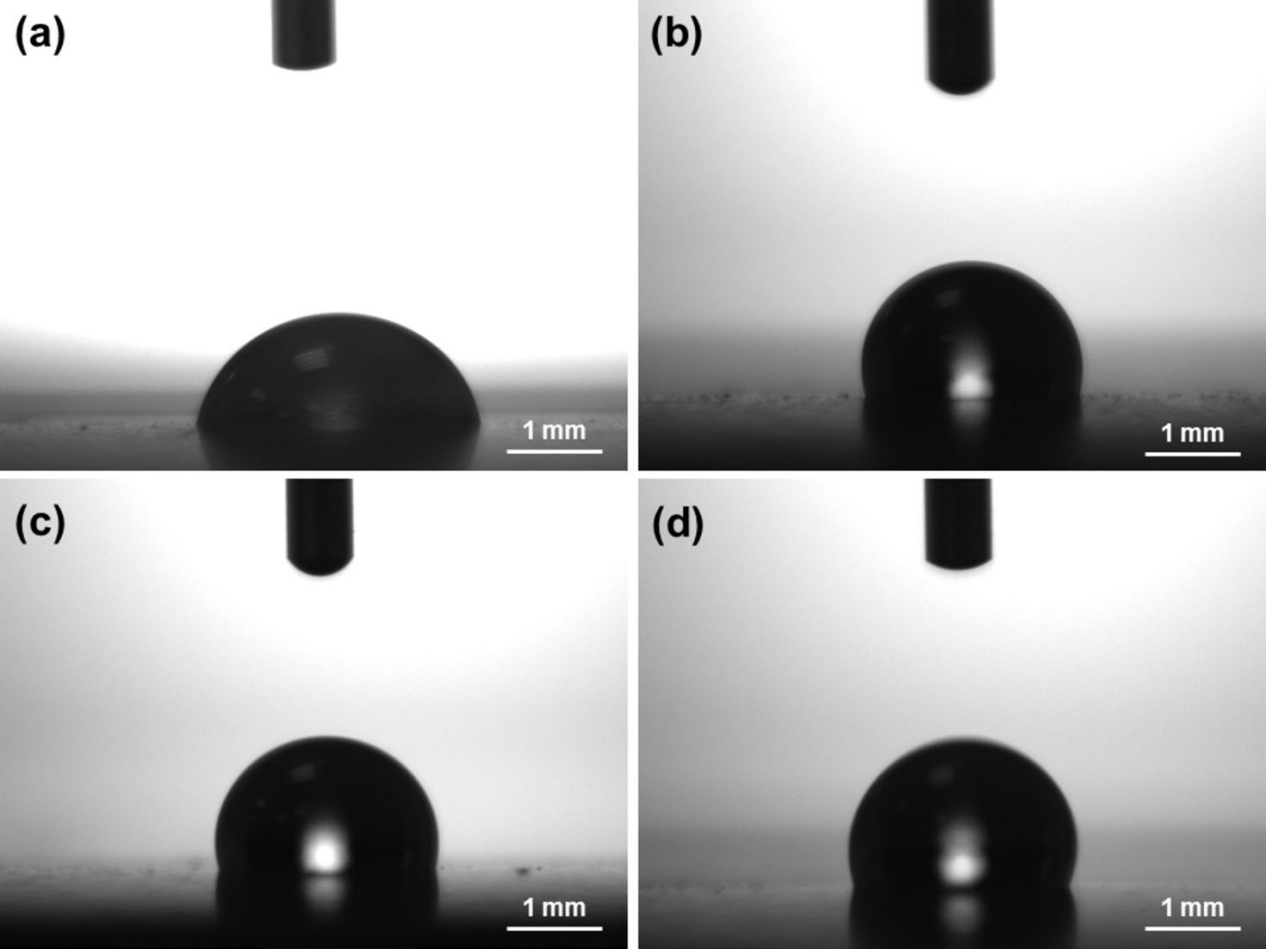
Images taken during the measurement of the contact angle of a water droplet on the surface of different coatings: (**a**) Ni coating, (**b**) Ni/PTFE produced with IPA, (**c**) Ni/PTFE produced with ethanol, and (**d**) Ni/PTFE produced with ethanol interrupted at 5 A·min.

Hydrophobicity of the Ni/PTFE coatings was already observed during the coating deposition, when the plating solution was expelled from the coating area. The contact angle of a water droplet resting on the coating surface was measured with an optical tensiometer (Fig. 8). The results (Table 2) show that coatings that failed to co-deposit PTFE resulted in hydrophilic surfaces, with contact angles < 90°. On the other hand, Ni/PTFE coatings present hydrophobic contact angles of 104–114°.

**Table 2:** Contact angle of a 5 μl water droplet resting on the surface of different coatings after 30 s. The deposition parameters of the coatings are listed on the left-hand side of the table.

Deposition parameters				Hydrophobicity
Suspension agent	Brush material	Current density (A/dm^2^)	Deposition time (A·min)	Contact angle (^o^)
None	White	124	24	78 ± 3
None	Red	75	37	86 ± 8
IPA	White	37	38	110 ± 2
IPA	White	37	3	104 ± 4
Ethanol	White	37	38	104 ± 4
Ethanol	White	37	5	114 ± 3

#### Roughness and coefficient of friction

Due to the PTFE layer covering the globular structure of the nickel, the roughness R_a_ (arithmetical mean deviation of the profile) of the Ni/PTFE coatings (1.1–2.5 μm) is less than the single-layer, globular nickel which has values > 5.0 μm (Table 3). The double-layered coatings from early stages of growth (3 and 5 A∙min) are significantly smoother than the thicker coatings of both alcohols.

**Table 3:** Coefficient of friction and average roughness R_a_ measured in several Ni and Ni/PTFE coatings.

Coating	Suspension agent	Deposition parameters			Characteristics	Roughness R_a_ (μm)
		Brush material	Current density (A/dm^2^)	Deposition time (A·min)	Coefficient of friction	
Pure Ni	–	Red	37	–	0.156 ± 0.006	–
	–	Red	75	–	0.191 ± 0.003	5.0 ± 0.8
	–	White	75	–	0.269 ± 0.006	11.6 ± 1.6
	–	White	124	–	0.247 ± 0.012	13.7 ± 2.0
Ni/PTFE	IPA	White	37	38	0.108 ± 0.001	1.8 ± 0.4
	IPA	White	37	3	0.096 ± 0.001	1.2 ± 0.3
	Ethanol	White	37	38	0.117 ± 0.003	2.5 ± 0.5
	Ethanol	White	37	5	0.107 ± 0.004	1.1 ± 0.2

The coefficient of friction (COF) of the coatings was measured against a chrome steel ball under continuous increasing load from 5 to 100 N. No delamination of the coatings was observed. Single layer nickel coatings present a linear increase of tangential force for the range of normal force applied, giving a COF of 0.16–0.19 for the smooth coatings and up to 0.27 for rough ones. On the other hand, the Ni/PTFE coatings only show a linear increase of tangential force beyond a normal load of 16–18 N; for lower loads (from 5 to 16–18), the tangential force actually decreases until a minimum is reached. The COF of 0.11 ± 0.01 shown in Table 3 corresponds to the 16–100 N stretch and is significantly smaller than with the nickel coatings. For lower loads, the value of COF may be lower than 0.10.

#### Corrosion resistance

**Fig. 9:**
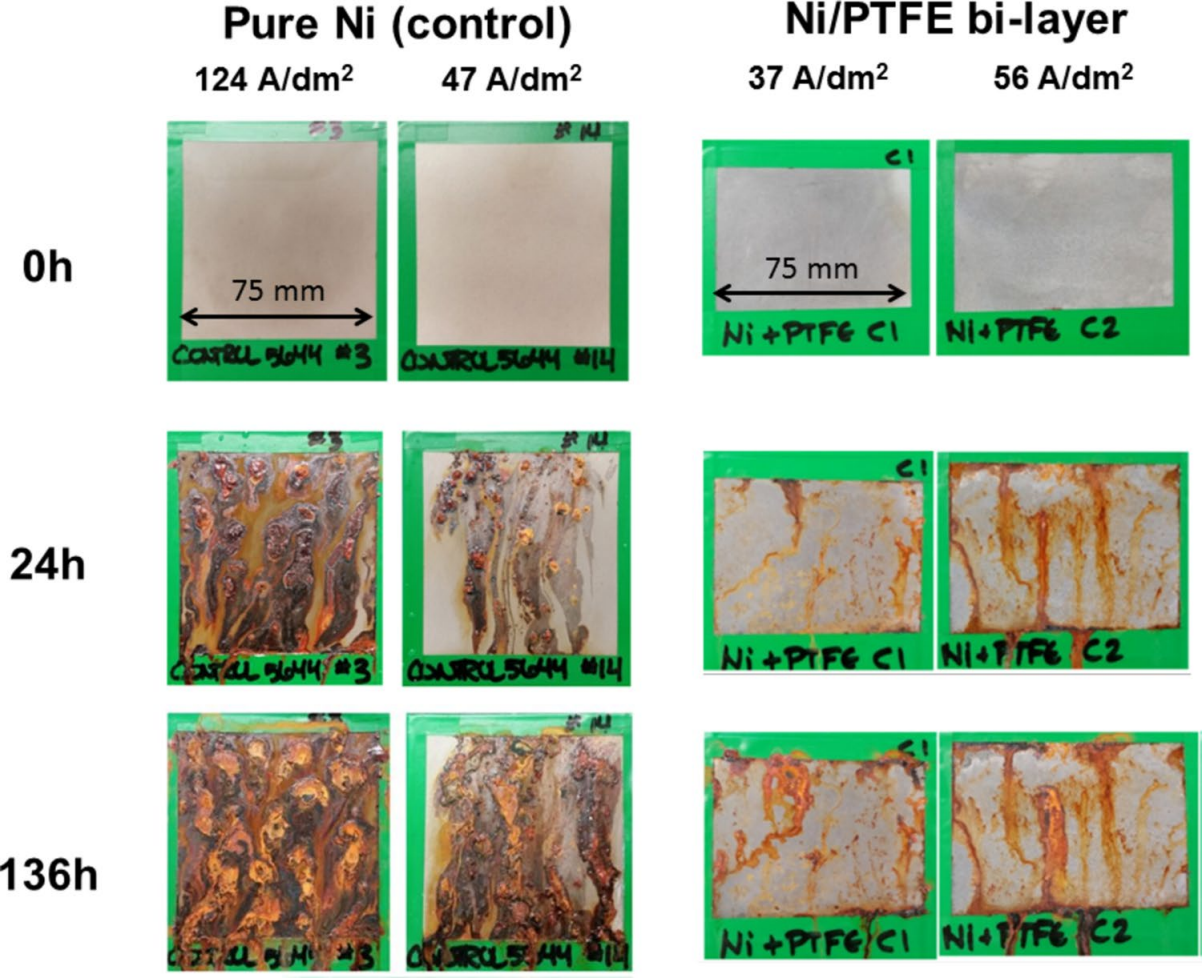
Comparison of Ni and Ni/PTFE coating surfaces after salt spray testing for 136 h.

Corrosion resistance of the Ni/PTFE coating was tested by exposing the surface of two samples to a salt spray test for 136 h. Two nickel high speed coatings were also tested as control samples. The samples were examined after 24, 48, 72, and 136 h; images of the results are given in Fig. 9. The single-layer nickel control samples show acute corrosion and red rust after 24 h, and the surfaces at the end of the test are greatly deteriorated, completely covered by red rust. The Ni/PTFE coating better protects the steel substrate from corrosion; although red rust is also present after 24 h, it is in smaller quantities and its condition does not significantly worsen over the next 112 h.

## Discussion

The addition of chemical compounds to produce the PTFE suspension in aqueous plating solutions is unavoidable. The attempts to produce composite Ni/PTFE coatings without any suspension agent were difficult, unsuccessful or produced non-homogeneous results. From the different compounds tried, the cationic surfactant CTAB produces the best quality suspension in which all the PTFE particles are easily suspended, agglomeration is not evident and the PTFE particles remains in suspension after the coating deposition finishes. These findings are in line with diverse studies^10^, which favour the use of CTAB^7^ and other cationic surfactants^12–14^ for tank electroplating because of the surfactants sharing the ionic charge (positive) with the ions being deposited. Despite this, the co-deposition of PTFE on the coating was not accomplished. This can be explained by the major differences between the brush plating process from the present study and the tank plating involving CTAB performed by Iacovetta et al.^7^. The present study uses current densities 3.7–12.4 times higher, a more neutral solution bath with different chemical composition, permanent anodes, and a brush that wipes the surface being coated. The differences persist when comparing with studies using other cationic surfactants^12,13^, except in the case of Wang et al.^14^ in which they use a similar current density (50–90 A/dm^2^) for jet electroplating. Any of these differences could have negative effects on the co-deposition of PTFE, although current density is likely to be involved if the effects of surfactant concentration on the solution conductivity are considered. Changes in solution conductivity have been reported and depend on the relative amounts of surfactant and particles present in the solution^8^. In our study, large amounts of CTAB had three effects: i) voltage had to be increased to obtain the same current densities (implying a decrease in solution conductivity), ii) it was detrimental to the electrodeposition process, and iii) it changed the morphology of the deposited coating. In tank plating, the effects of CTAB might not be so severe because of the lower current densities used; in brush plating, the presence of the surfactant alters the deposition process. Comparisons with the literature are difficult, as no publications of CTAB-suspended PTFE particles for brush plating were found.

The use of alcohol has advantages and inconveniences. From the point of view of suspending PTFE, the use of alcohol is less appealing, as it produces particle agglomeration and a noticeable quantity of particles drop out of the suspension after the deposition process, probably due to alcohol evaporation. Nonetheless, alcohols proved most effective in the co-deposition of PTFE, with both IPA and ethanol producing very similar results. Again, comparisons with the literature are difficult as, to the best of the authors’ knowledge, the use of alcohols for PTFE suspension and co-deposition has not been reported. Moreover, usual co-deposition of PTFE occurs as a dispersed phase within a metal matrix for both tank and brush electroplating^7,10–14^; in this work, a PTFE compact layer has been created by electroplating processes, which has not been reported yet. The coating presents a bilayer-like, partially intercalated structure of compact layers of PTFE (top) and nickel (bottom). The nickel sublayer retains its traditional globular structure, which intercalates on the central area with the PTFE covering the valleys, and prevents the structure from becoming clear-cut bilayer structure of two flat, parallel deposits. The production of a compact PTFE top layer may be explained by comparing brush plating to the process used to produce PTFE coatings. A detailed description of the PTFE coating process is given by McKeen^25^ and can be summarised in three stages. The first stage is surface preparation, when the surface to be coated is cleaned and can be roughened for optimum adhesion, as the coating bonds mechanically on the substrate. Then, a mix of PTFE with solvents and additives in liquid or powder form is sprayed on the rough surface. The third and final stage is baking the coating so the binder evaporates and the powder melts together. The baking subdivides into different sub-stages, each with increasing temperature and different times, starting at 149 °C and finishing with a stage at 371–438 °C. The two first main stages are emulated during the brush plating process: a rough Ni layer is first deposited, and then PTFE particles are likely to be co-deposited on the Ni surface. However, the third stage (particle fusion) is the main difference between both processes; whereas the traditional coating process heats the coating above the melting point of the thermoplastic PTFE (327 °C), such temperatures cannot be reached in non-pressurised aqueous solutions. Although the solution temperature increased after the electrodeposition (common in nickel deposition), unusual amounts of water evaporation were not detected. The use of alcohol does not seem to be the cause either, as alcohols have not been reported to dissolve PTFE and a partial Ni/PTFE was produced in one sample that did not use alcohol. However, that the alcohol may be playing a role cannot be ruled out completely, as no other publication has used them for Ni/PTFE co-deposition. The energy to fuse the particles together may come from the potential differential applied: PTFE particles are not conductive, thus any electric current passing through them causes localised heating. However, it is unknown if the temperature increase is high enough to cause melting. Clearly, incorporating PTFE powder into the nickel plating is challenging, forming a purely nickel coating. During the plating process with a solution system that includes IPA and ethanol, as the plating time extends and temperature rises, much of the IPA and ethanol would evaporate. This evaporation could also cause the PTFE powder in the solution to gradually accumulate and adsorb onto the substrate interface, forming a distinct double-layer structure.

The samples showing early deposition stages contributed to the understanding of the growth of these coatings. The amount of charge applied to the coatings (A·min) can be used as reference: the IPA sample was interrupted at 3 A·min, earlier than the ethanol sample interrupted at 5 A·min. The surface and cross-sectional images show that there is an initial stage where only Ni is depositing. At some point, formation of the PTFE layer starts in isolated Ni layer valleys (Fig. 5b) and then grows, covering more and more of the nickel globules (Fig. 6b shows only the tips) and joining the isolated valeys together until all the globules are completely covered by PTFE. There are no instances of Ni depositing on top of PTFE. The tips of the nickel globules are not very deeply covered in PTFE, which may explain why the nickel under-layer appears to continue growing (the nickel is significantly thicker when comparing Fig. 6b with Fig. 6d). Additionally, comparing Fig. 6b with d shows that the PTFE layer continues to grow after it completely covers the nickel. However, the coating growth is not linear with the total charge supplied to the system, which suggest growth rate is limited or, at least, non-linear: 5 A·min produces an average coating thickness of 7 ± 1 μm, while the 38 A·min gives 26 ± 6 μm instead of the 53 ± 8 μm that would correspond to linear growth predicted by Faraday’s law of electrolysis^26^, typical of electroplating a metal or alloy.

Microstructurally, the results reveal a small change in the nickel preferential orientation and crystallite size due to the presence of CTAB. For the Ni/PTFE coatings, the resulting patterns are just a combination of the Ni and PTFE peaks. Similar results were obtained by other studies^13,16^ despite producing a metal matrix composite coating instead of a double-layer structure. The outer layer of PTFE explains the hydrophobicity of the surfaces, with contact values within the 108–114° of smooth, unmodified, bulk PTFE^27,28^. By contrast, the behaviour of metal matrix composite Ni/PTFE coatings tends to be superhydrophobic, with contact angles typically 122–156° depending on the PTFE content^7,12,13^.

The outer PTFE layer also explains the decrease in coefficient of friction, and that higher loads would probably reveal the shallowest nickel tips, increasing the COF. Even the COF values obtained for higher loads are half of the lower published values for other Ni/PTFE coatings produced with brush plating^11,16^. Differences in COF between thick and thin coatings are observed, and can be explained by the slightly increased roughness of 38 A·min coatings. The increased roughness values can be attributed to preferential growth of the nickel tips, as the valley growth is hindered by thicker PTFE.

Finally, the PTFE outer layer improved the corrosion resistance of the coating compared with nickel control coatings, which is similar to results reported for MMC Ni/PTFE coatings^10,14^. The hydrophobicity of the outer layer may also contribute to this effect, as water is likely to be quickly expelled and material dissolution is reduced. The presence of red rust is to be expected, as the PTFE layer insulates rather than passivates and the shallower, thinly-covered nickel globule tips are likely permit access to the steel substrate where corrosion is rapid, as nickel high speed usually shows some degree of cracking^24^.

Future testing should be centred on understanding and explaining the mechanisms of the PTFE layer formation and on further characterisation of the intercalated, bilayer-like coating. Although the coating shows improved corrosion resistance, a thicker PTFE layer could further improve corrosion protection. Additional corrosion tests should be run to obtain complementary data to the salt spray testing, for example in different environments. The adhesion of the coating has not been tested, although the friction test did not produce coating delamination. Due to the low friction measured, the coating would likely resist dry, fretting and sliding wear, thus further testing should be undertaken to assess the wear resistance of the coating.

## Conclusions

Diverse chemical compounds were tested to produce a PTFE suspension in nickel high speed solution suitable for use in bush electroplating. The addition of CTAB produced a dispersed, homogeneous suspension but failed to produce the composite coating. Moreover, excess CTAB concentration in the plating solution proved detrimental for the plating process. More successful was the addition of IPA and ethanol, which allowed the co-deposition of PTFE. The resulting coating has a bilayer-like, partially intercalated structure composed of a rough nickel layer covered by a compact outer layer of PTFE that formed without the need of a baking stage. The coating growth does not follow the Faraday law of electrolysis as the thickness does not linearly increase with the total applied charge.

The presence of the outer PTFE layer significantly changes the properties of the coating. Characterisation of the Ni/PTFE revealed a hydrophobic surface with an average contact angle of 109 ± 5°, within the values of untreated PTFE. The friction of the coating against a chrome steel ball decreased 25–38% from the lowest value registered for a nickel coating, down to a COF of around 0.10. Reference values from hard chrome coatings show a COF of 0.16–0.23, significantly higher than Ni/PTFE. The outer polymer layer also significantly improved the corrosion resistance in a salt spray environment.

## Data Availability

The datasets generated during and/or analysed during the current study have been included in full in this published article. Further details are available from the corresponding author on reasonable request.
